# Supplementary figures and images for: Identification and characterization of miRNAs involved in cold acclimation of zebrafish ZF4 cells
Source: PLoS One. 2020 Jan 10;15(1):e0226905. doi: 10.1371/journal.pone.0226905 (PMC6953832; doi:10.1371/journal.pone.0226905)

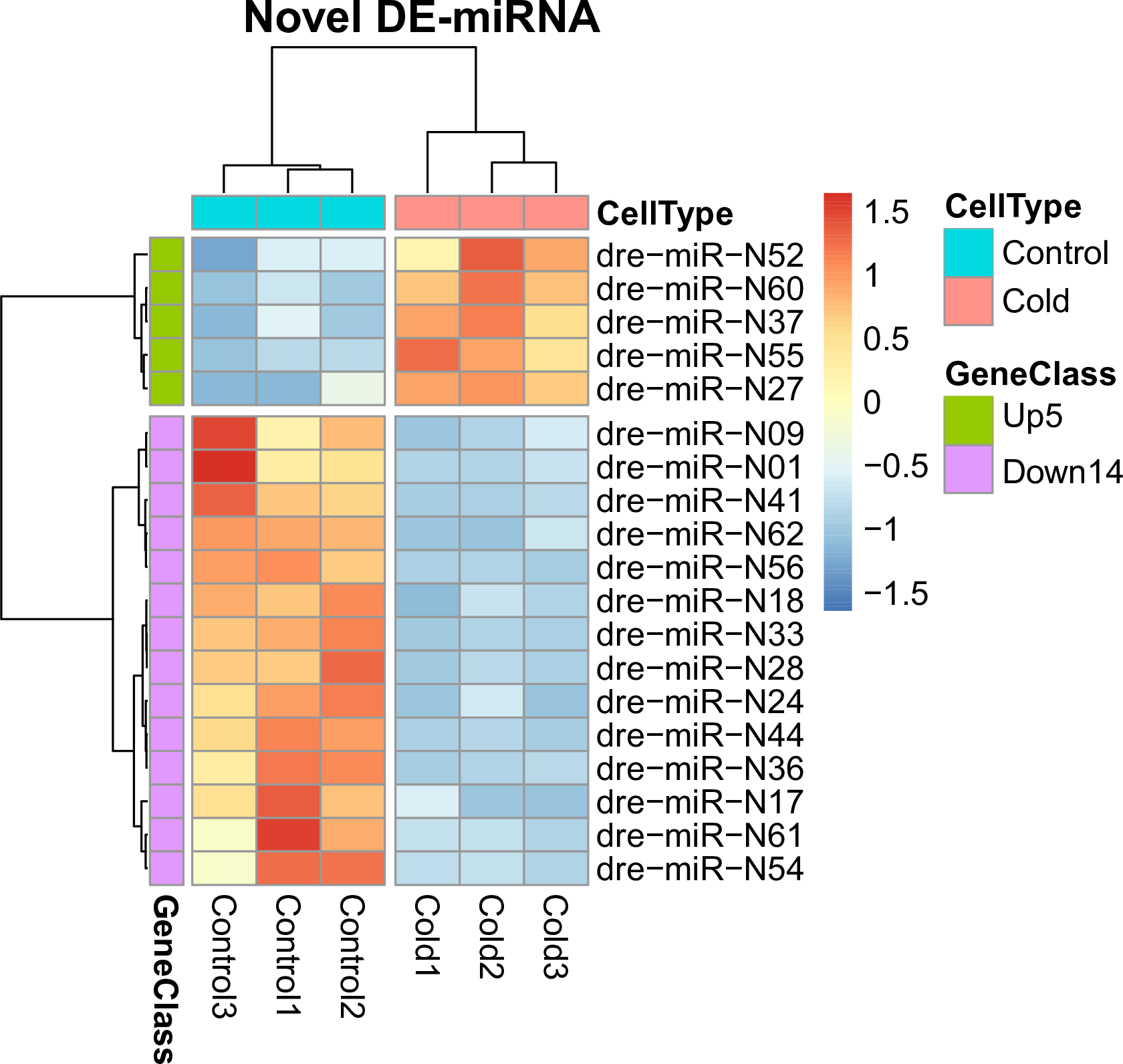

Supplement: S1 Fig — A heatmap was generated based on fold change values of novel DE-miRNAs to visualize the expression patterns of the cold responsive miRNAs. (TIF) [file pone.0226905.s001.tif]

NC-mimics

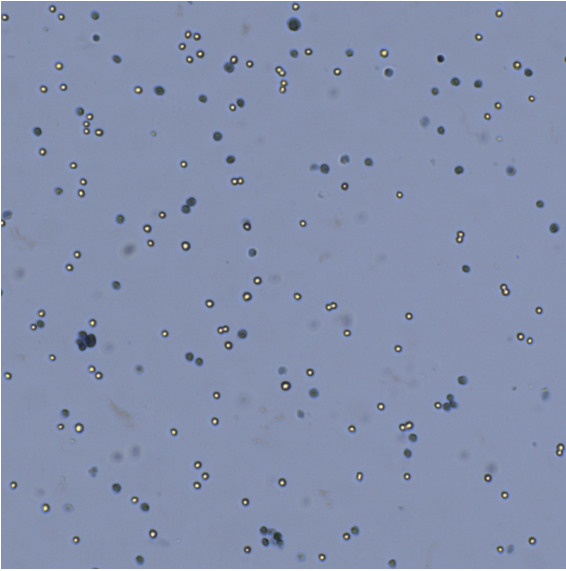

100-3p mimics

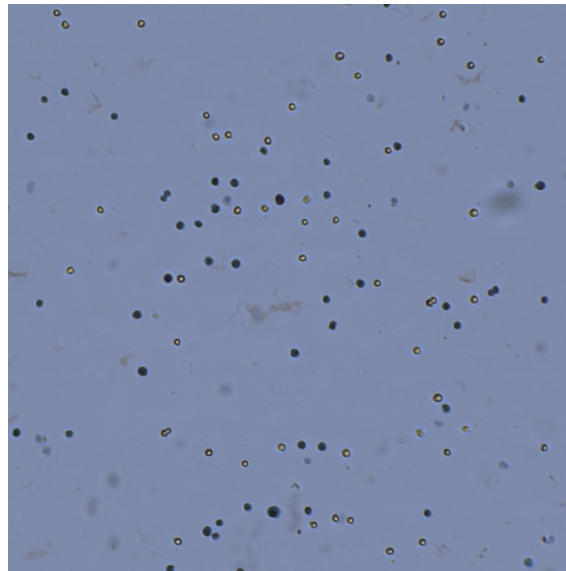

16b mimics

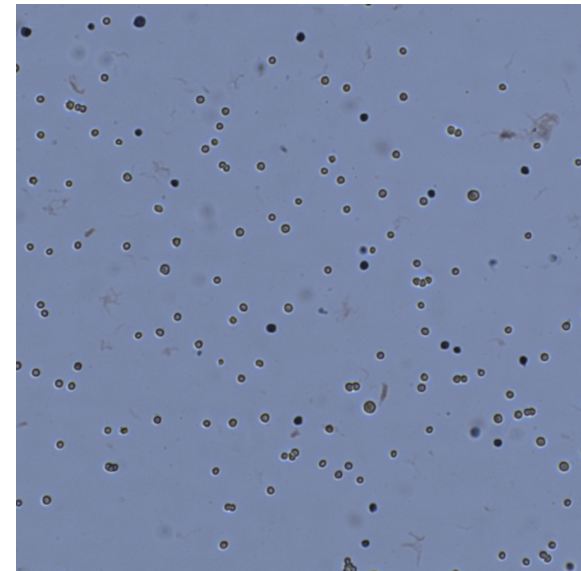

NC-inhibitor

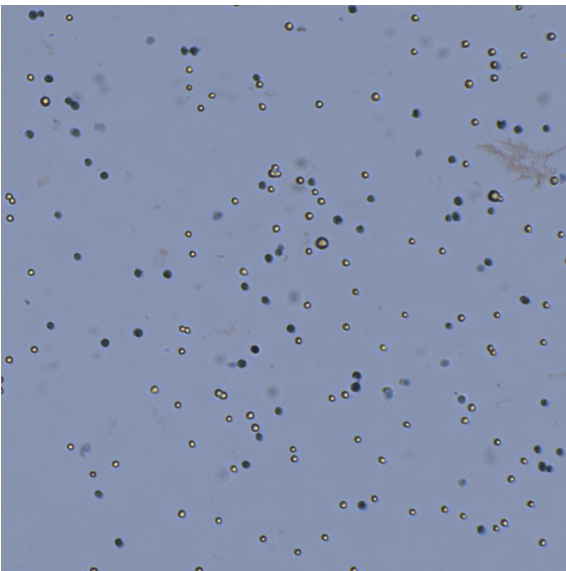

100-3p inhibitor

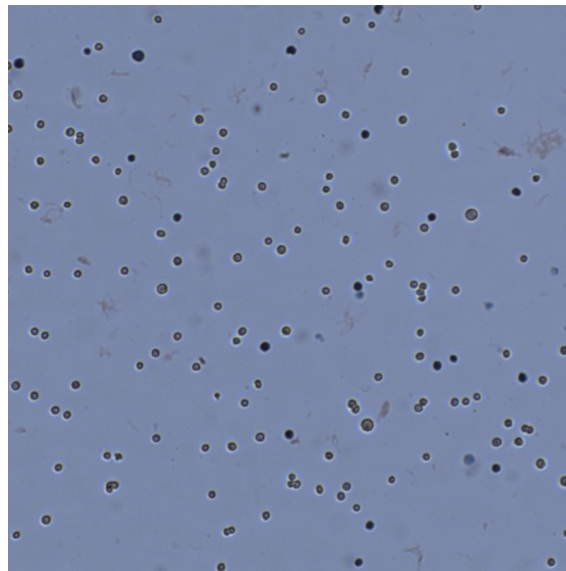

16b inhibitor

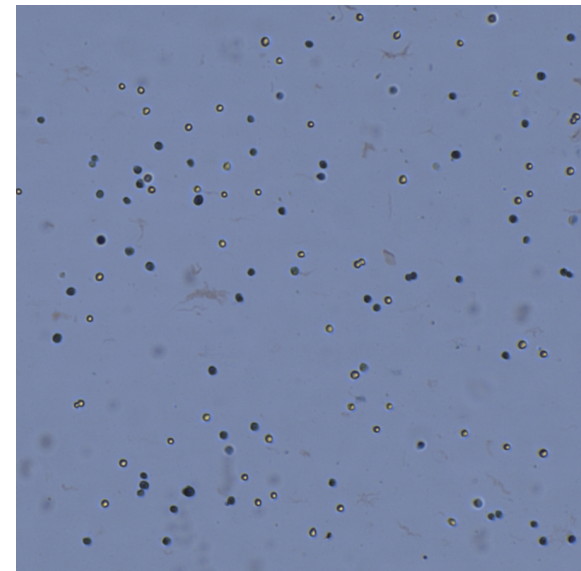

Supplement: S3 Fig — (PDF) [file pone.0226905.s003.pdf]
