# Supplementary material for: Identification and characterization of miRNAs involved in cold acclimation of zebrafish ZF4 cells
Source: PLoS One. 2020 Jan 10;15(1):e0226905. doi: 10.1371/journal.pone.0226905 (PMC6953832; doi:10.1371/journal.pone.0226905)
Supplement: S2 Fig — (PDF) [file pone.0226905.s002.pdf]

```
Position 1125 - 1146
3’ ctaTGGATATCTATGTTCGAAc 5’   dre-miR-100-3p
      ||||: || |||||||||
5’ gaaACCTGAAGTTACAAGCTTg 3’   atad5a

Position 1188 - 1208
3’ ctatgGATATCTATGTTCGAAc 5’   dre-miR-100-3p
        ||:|||:| |||||||
5’ gggggCTGTAGGT-CAAGCTTg 3’    lamp1

Position 327 - 349
3’ ctATGGATATC-TATGTTCGAAc 5’   dre-miR-100-3p
     | ||||||| : ||||||||
5’ ctTTCCTATAGTGGACAAGCTTg 3’   cyp2ae1

Position 2113 - 2134
3’ gaGGTTATAAATGCACGACGAt 5’   dre-miR-16b
     |: | : ||||||||||||
5’ ttCTTAAGGTTACGTGCTGCTa 3’   btbd9

Position 3251 - 3273
3’ gaGGTTATA-AATGCACGACGAt 5’   dre-miR-16b
     ||:| || ||::||||||||
5’ ctCCGAGATCTTGTGTGCTGCTc 3’   tnika

Position 6739 - 6761
3’ gaGGTTATAAATGC-ACGACGAt 5’   dre-miR-16b
     ||| |:|||| | |||||||
5’ gcCCATTGTTTAGGCTGCTGCTa 3’   atxn7

Position 1154 - 1176
3’ gaGGTTATA--AATGCACGACGAt 5’   dre-miR-16b
     |||:|:|   ||| |||||||
5’ atCCAGTGTACATAC-TGCTGCTg 3’   rilp
```
