# Supplementary material for: Identification and characterization of miRNAs involved in cold acclimation of zebrafish ZF4 cells
Source: PLoS One. 2020 Jan 10;15(1):e0226905. doi: 10.1371/journal.pone.0226905 (PMC6953832; doi:10.1371/journal.pone.0226905)

The secondary structure of novel miRNAs

GAAAGATGTAACCATTGACTTT dre-miR-N01

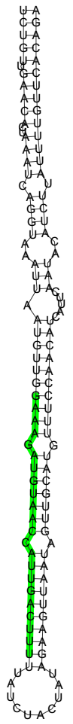

TTCACTGTGGCGGAAATGACC dre-miR-N02

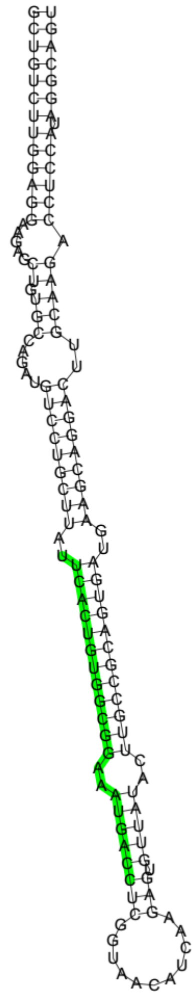

TCCATCAGTCACGTGACCTACC dre-miR-N03

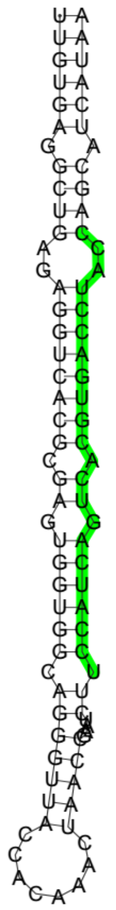

ACATGGAAGCATTTCTGATTT dre-miR-N04

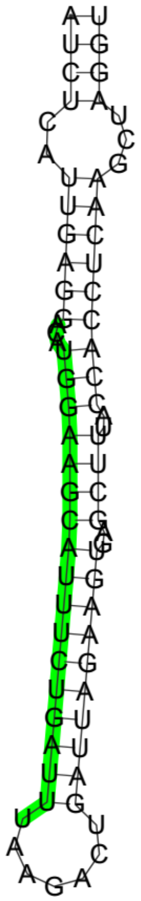

TAAAAAGCCTTTATTTACATGAC dre-miR-N05

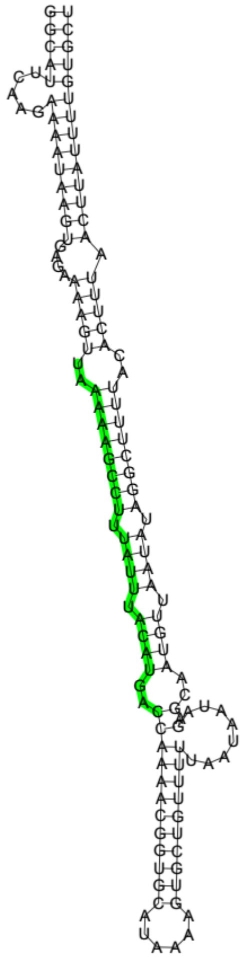

TTCTGTTTGGTACGCTTTTATGG dre-miR-N06

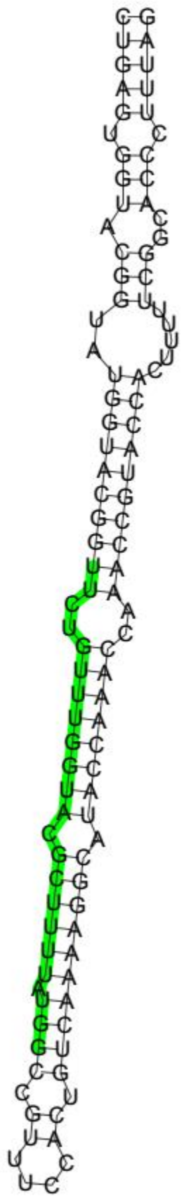

TATGAAAGTCAATGGTTACCGT dre-miR-N07

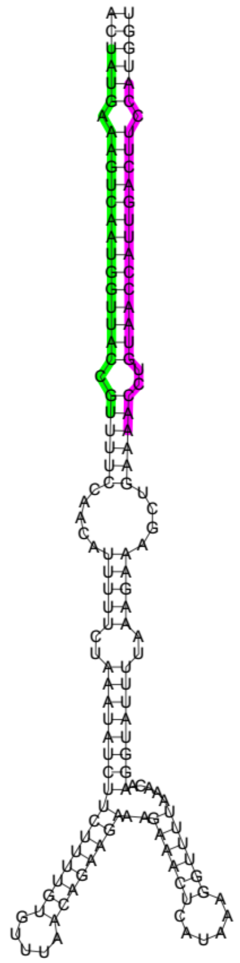

AGTTTTGCTGGTTTGCTGTC dre-miR-N08

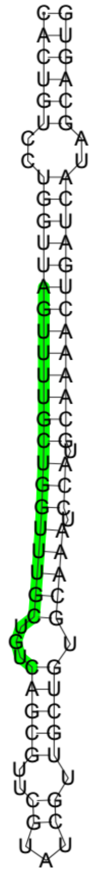

CTGTACGGTTCAGTTTGGTATG dre-miR-N09

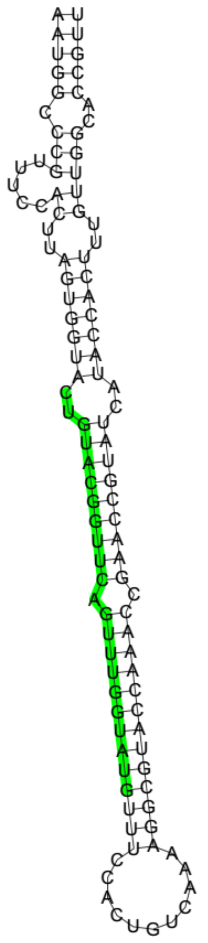

TTGGAGAAAGGATTTCCTGAAC dre-miR-N10

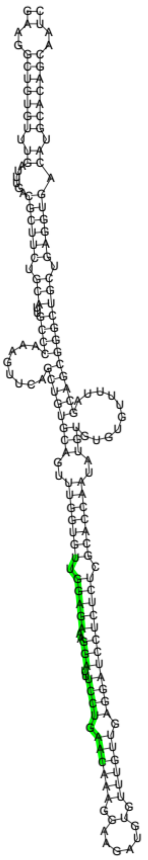

CTGCAGGGCGTCTCTGGAG dre-miR-N11

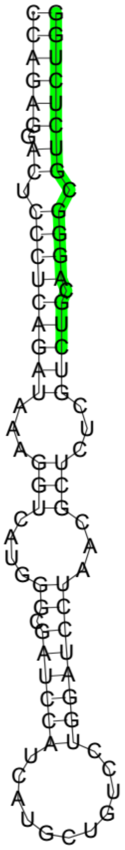

TTGCTGTTATTTTCGTAGGTGT dre-miR-N12

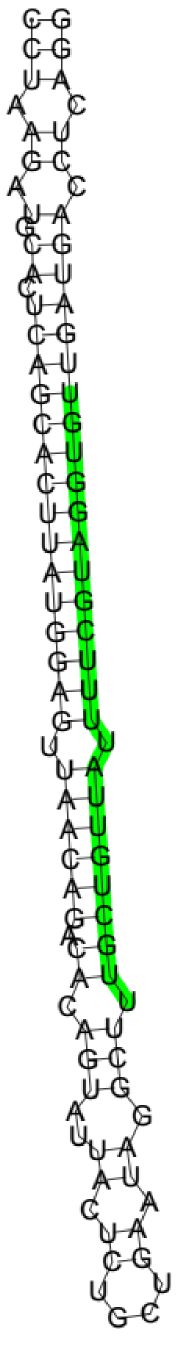

TTTGAGAACCTGTAACCATTGA dre-miR-N13

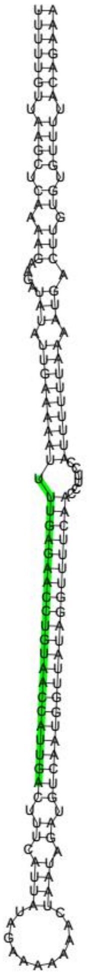

TTTCTGTTGTTTTCATGAGGCA dre-miR-N14

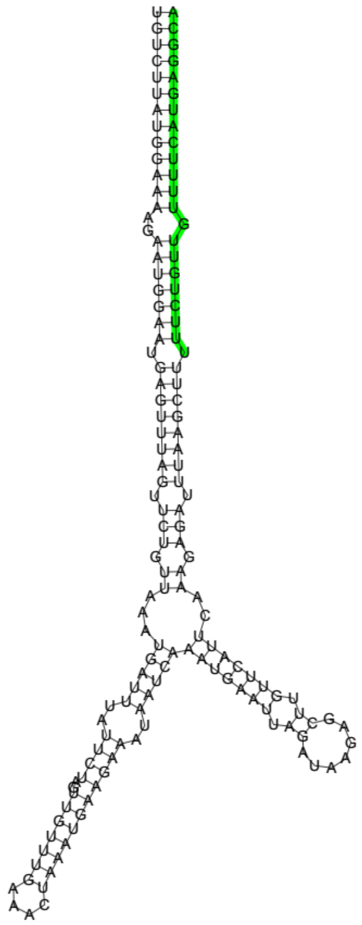

TCCACAATTCAGTTTGGCAATG dre-miR-N15

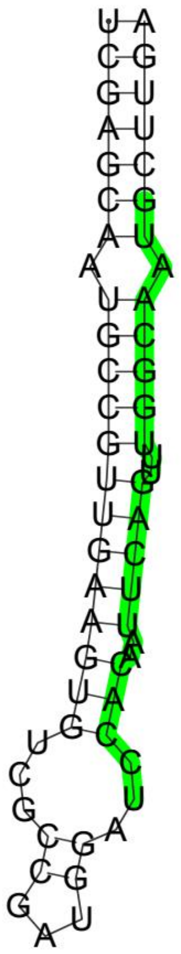

CCCGGTGTCCTGCATAGTTTAG dre-miR-N16

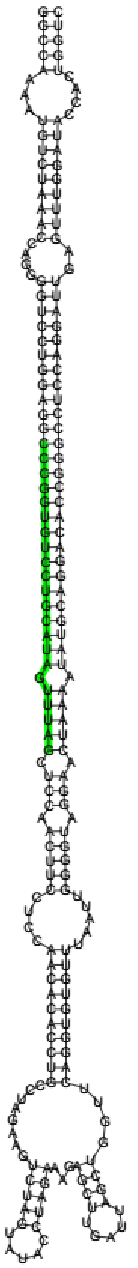

CTCCAACCTTGATCAAGCAGCT dre-miR-N17

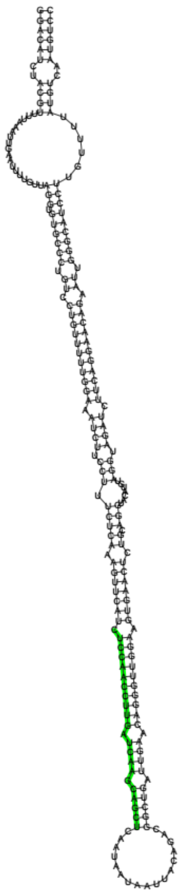

AACAACATGTCACTCTAGGCT dre-miR-N18

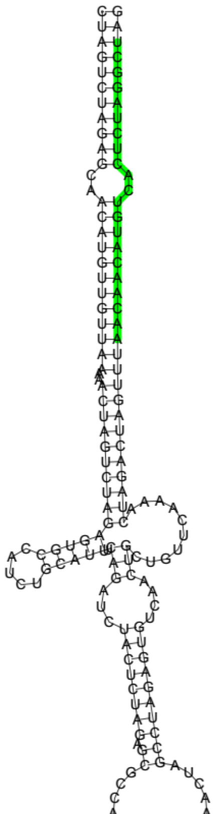

GAGGAATTGACTTTGACACTCCT dre-miR-N19

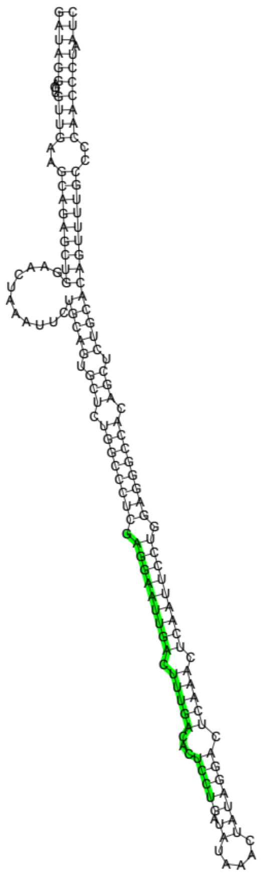

AATGTATTTTTGGGCTGAGGGA dre-miR-N20

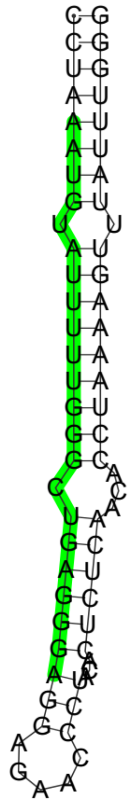

ATGACTCAAACCCGGGGACTCG dre-miR-N21

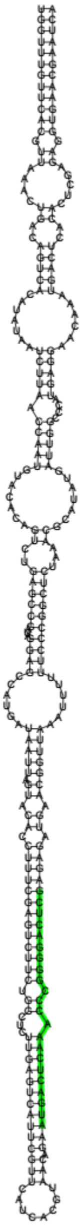

ATGATAATGATGATGATGATGCT dre-miR-N22

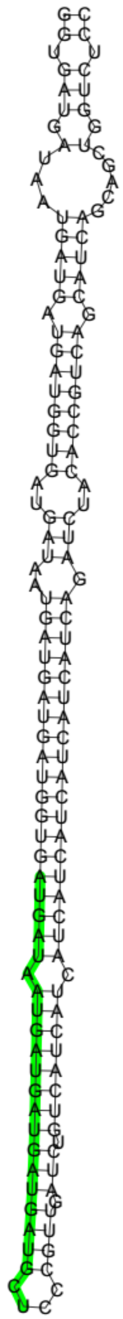

ATTTTCAATTCTGTGTGAACT dre-miR-N23

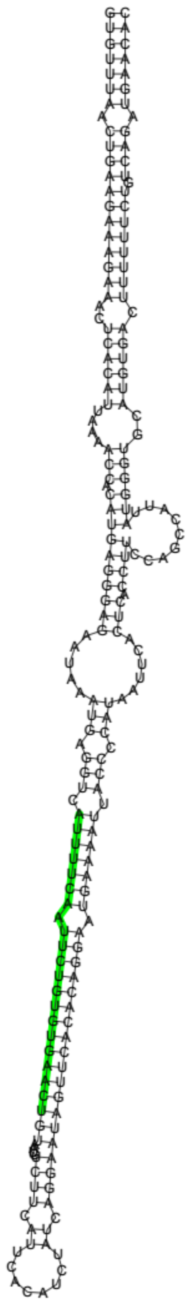

GTGACCAAAATCAAACGTCTGAT dre-miR-N24

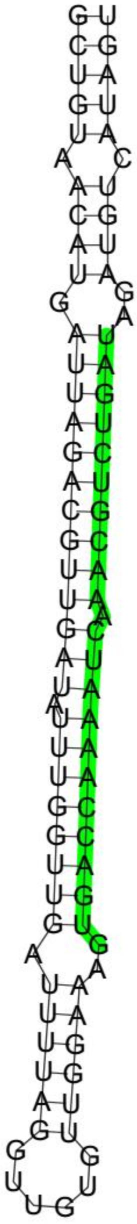

ACAACCTTGATCAAACGCAACT dre-miR-N25

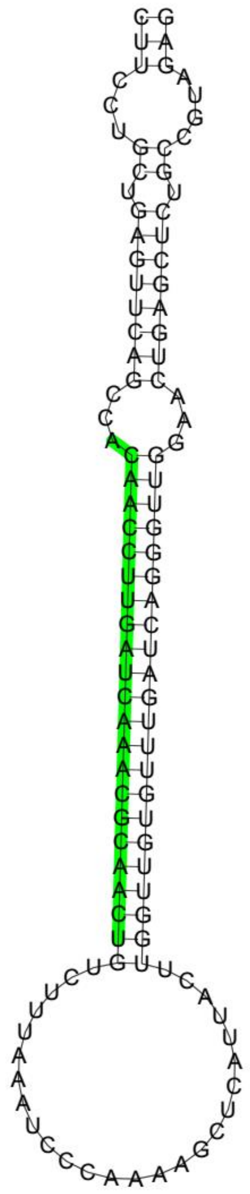

AGTCTGTAGAGCATGAGAC dre-miR-N26

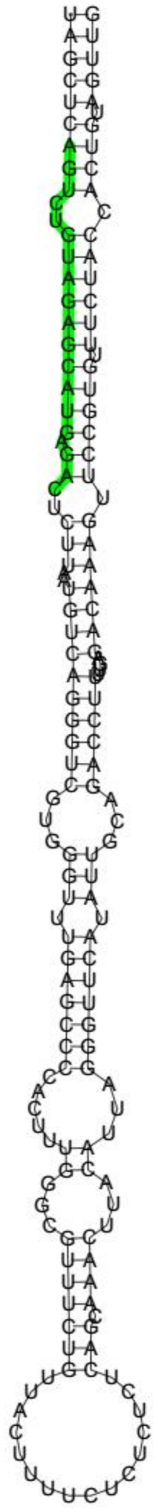

ACCTTAGAGGAGAACGACGGCCC   dre-miR-N27

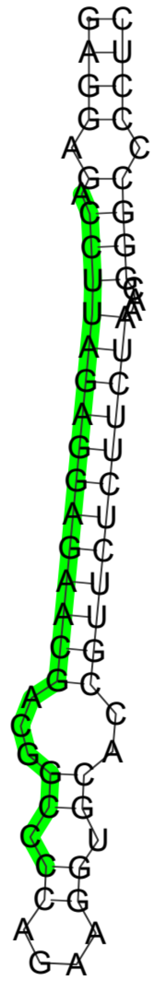

TGCAGGACACCGGACCTCCAGT dre-miR-N28

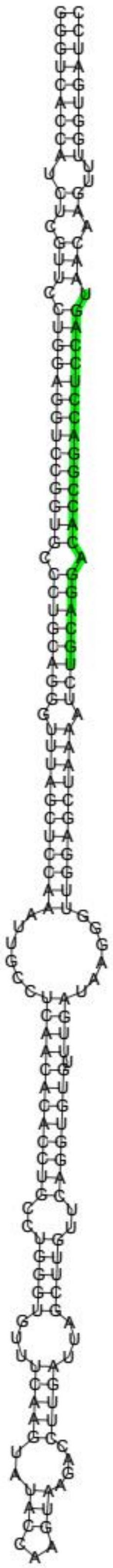

TTCGAAATACCTTAGTCACATG dre-miR-N29

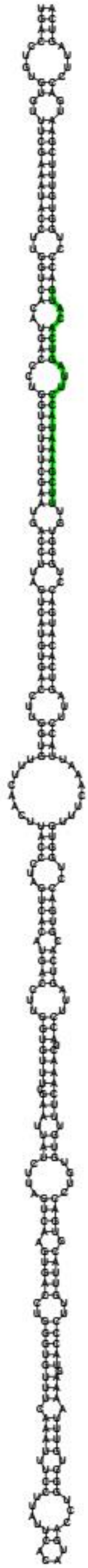

TGCAGGACACCGGACCTCCAGGT dre-miR-N30

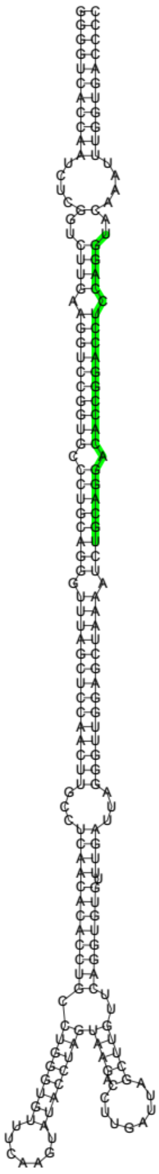

TAGGGTATCAGACTGGTGT dre-miR-N31

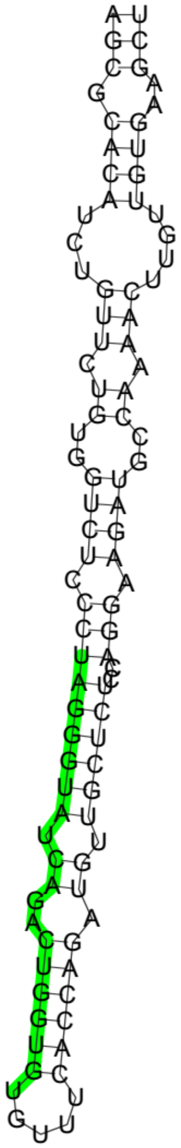

CTCGGATCATCTGTTTTCCGGC dre-miR-N32

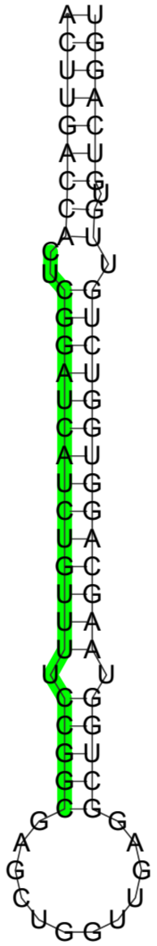

TAACGTTTCGAGCCCACTGACTG dre-miR-N33

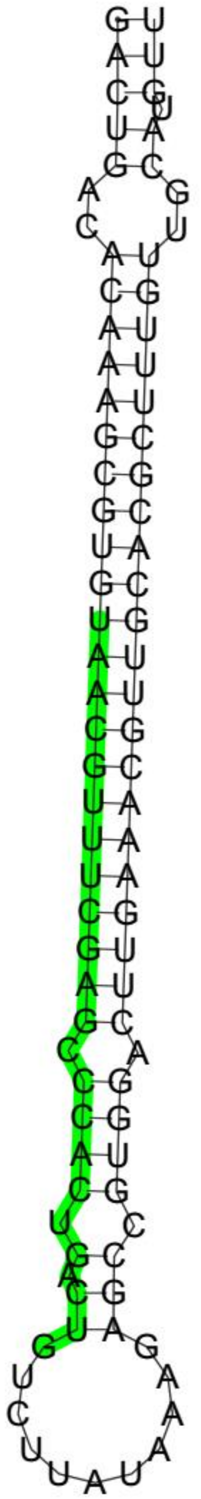

AATAGACAGATTCGAAAGACT dre-miR-N34

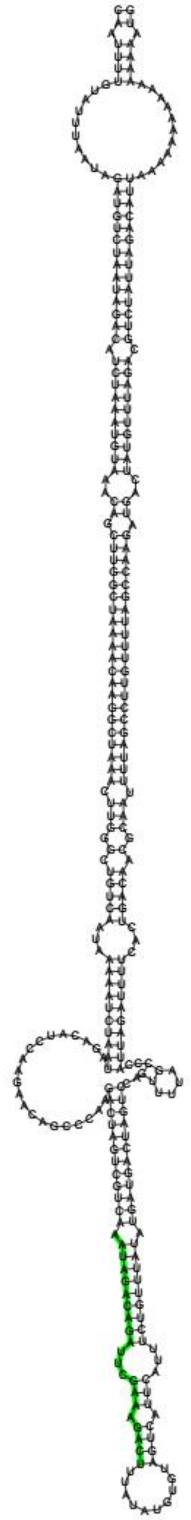

TGTGGAAGTCAATGGTTACAT   dre-miR-N35

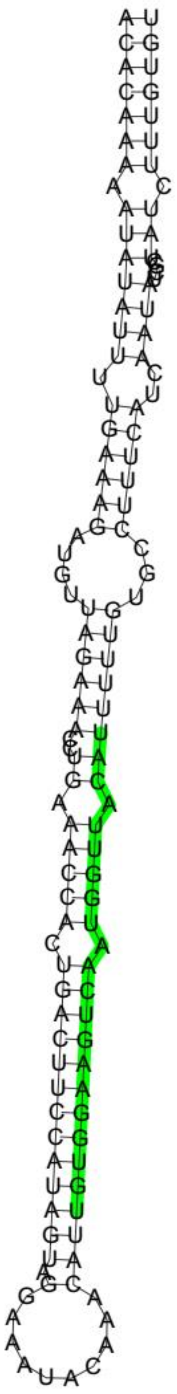

ATGACTCAAACCCGAGGACTCA dre-miR-N36

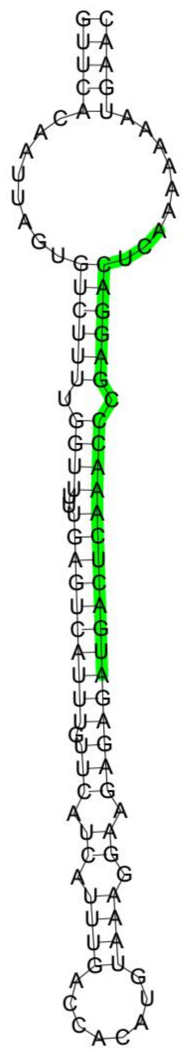

TCGATGCTGGATTTCTCAACCT dre-miR-N37

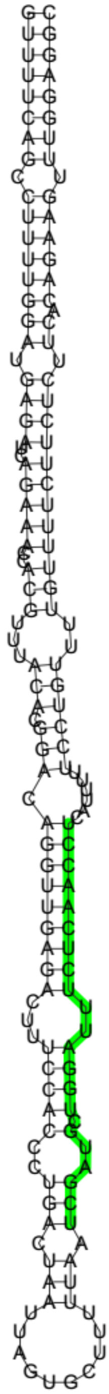

TGTGAGCTGGTTTATACTGACCT dre-miR-N38

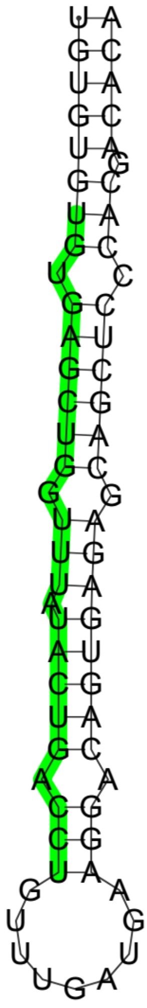

TCACCATTGACTTCTGTAGT dre-miR-N39

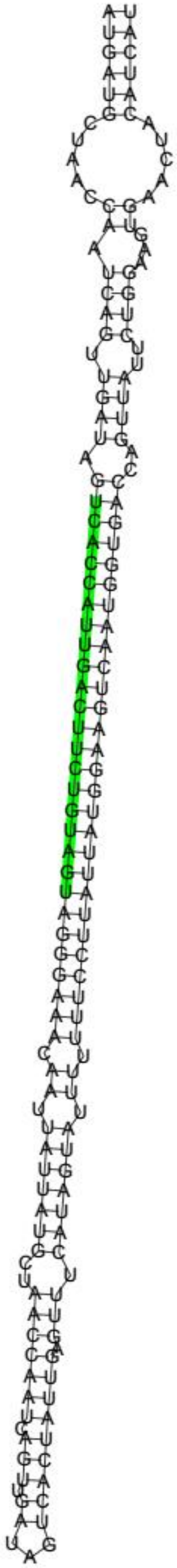

AGACTAGTAGCCATTGAGATCT dre-miR-N40

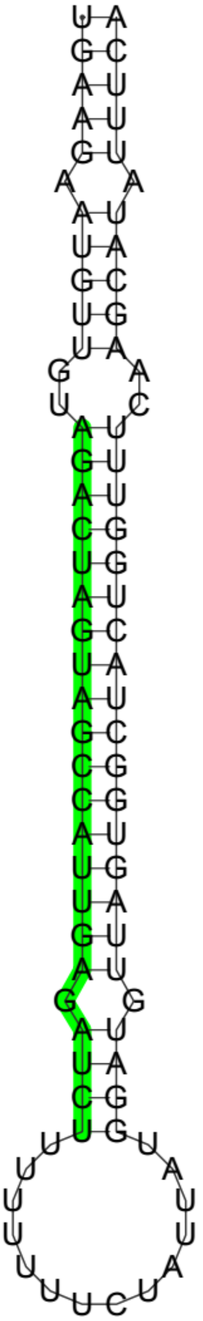

AGCTGGTGTCCTGCAGAGTTT dre-miR-N41

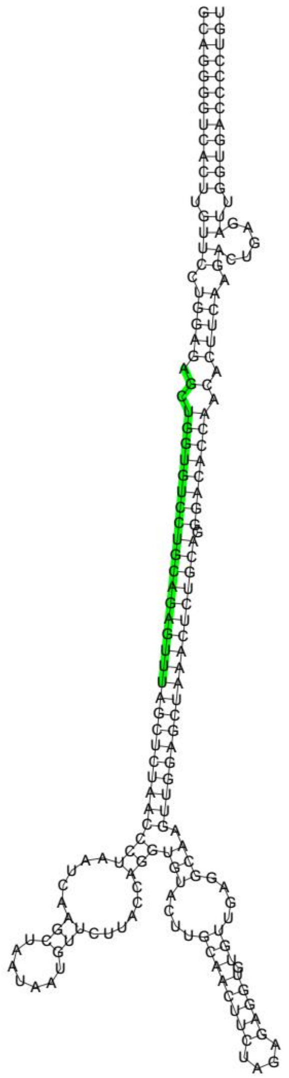

GAAATTGATATTGATAACTGGTC dre-miR-N42

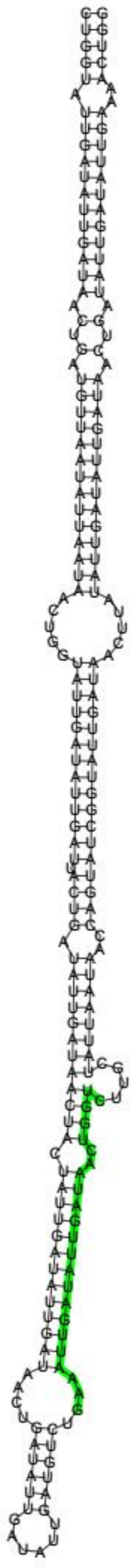

ATCGCTGGTAATCGTGACAGGT dre-miR-N43\

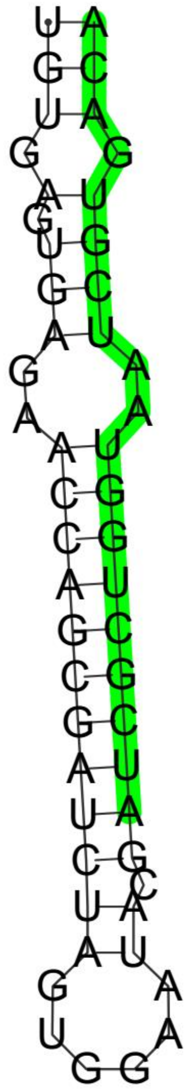

TTTCCGGCCAGATGCGGCGGCCG dre-miR-N44

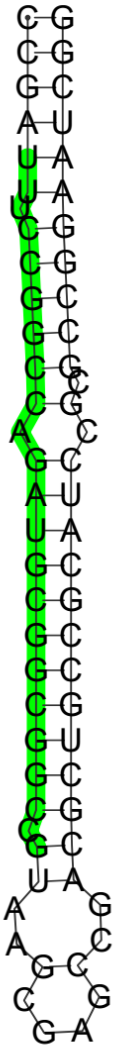

AAGTTCTGTGGTCCACTCTGGC dre-miR-N45

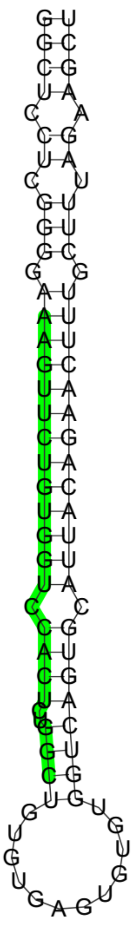

TATTGTTGTCCAAACTCGGTCC dre-miR-N46

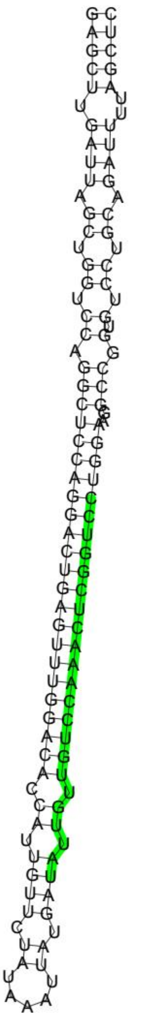

TTCACTGTGGCGGAAATGACCT dre-miR-N47

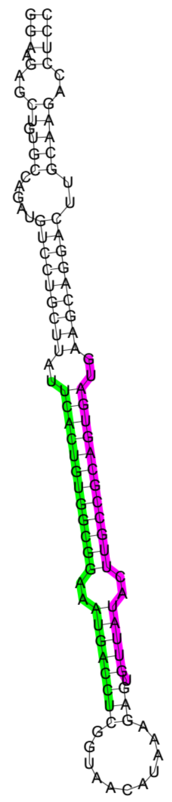

TCTGGTCCTGTCTTGGCTTAC dre-miR-N48

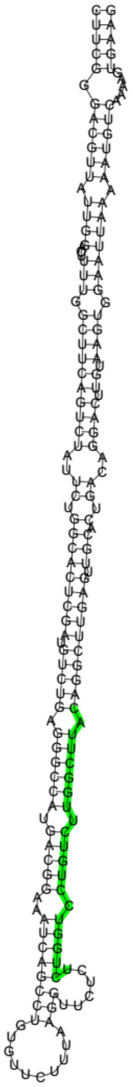

CTGTGTTCGCGCTCTCCCCTG dre-miR-N49

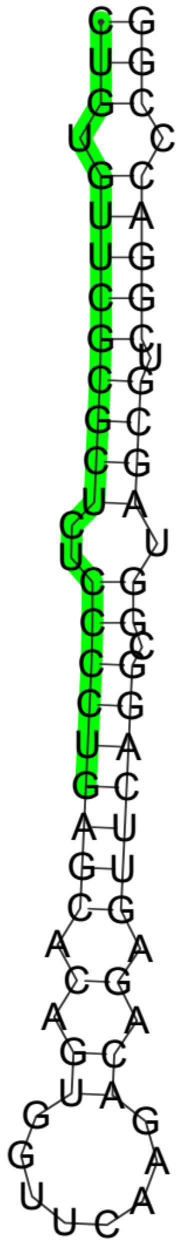

AGGGTTATGATTCTCGCT dre-miR-N50

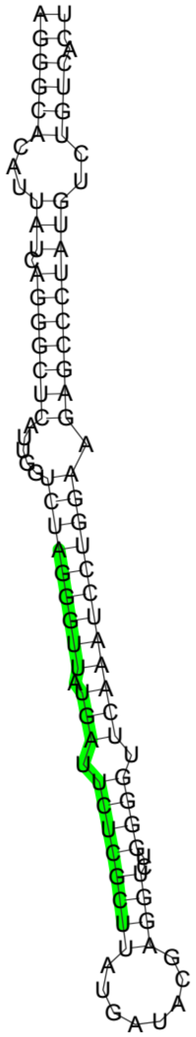

ACGCTTCTCTTGGCTGTCTGATT dre-miR-N51

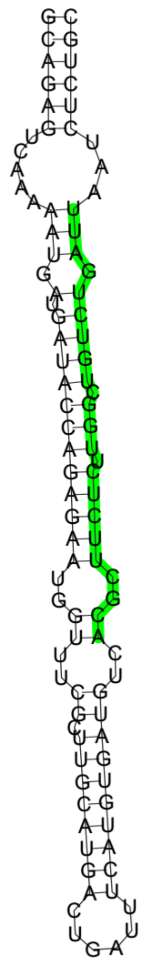

CCCGGCGCCCCCCGGATGCCCT dre-miR-N52

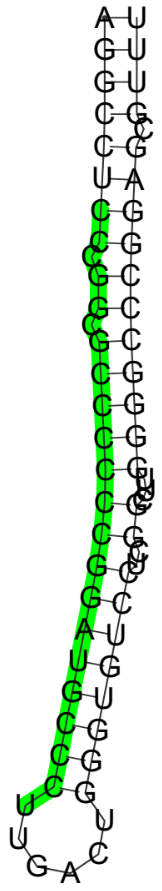

GATGCCATCGAACATTGCTGAT dre-miR-N53

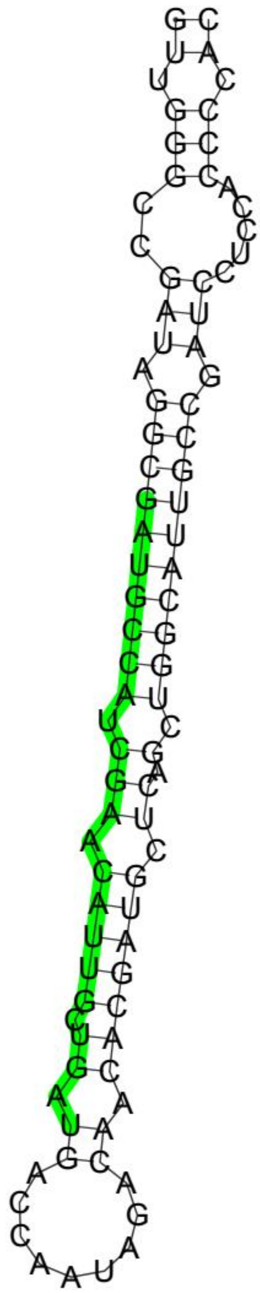

TAAGTCAGGGGTGTCTAAACT dre-miR-N54

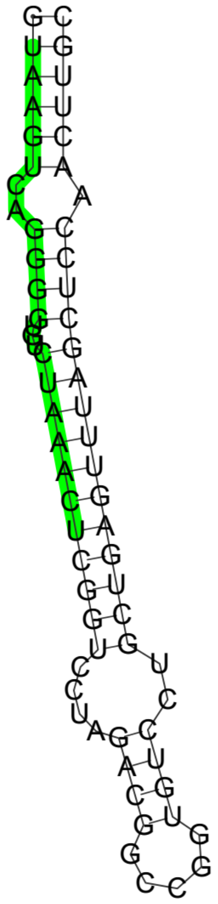

ACTGCTGATAAGCCTGTGGGGC dre-miR-N55

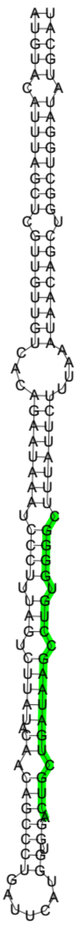

ATGACTCAAACTTGAGGACTT dre-miR-N56

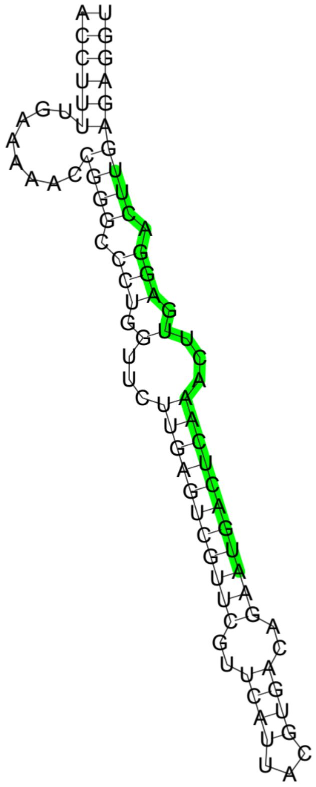

CAGTACACCGGACCTCCAGGA dre-miR-N57

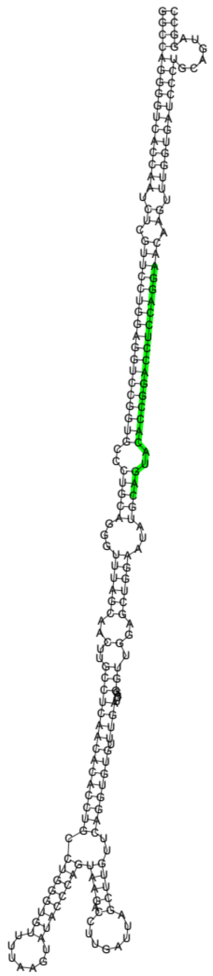

GTCACGGTGGCCGAGAGGTTAA dre-miR-N58

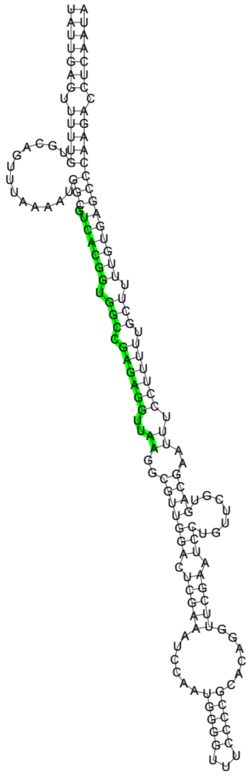

TGATGCATCATCTATAGTGATTG dre-miR-N59

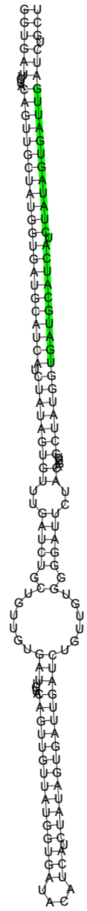

CAGCACAAGGCTACGCTCTTCTdre-miR-N60

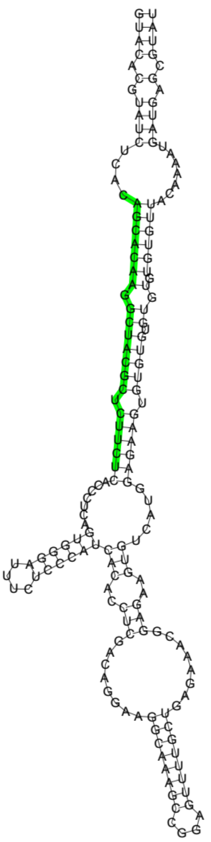

TTACAATTAAAGGATATTTCTT   dre-miR-N61

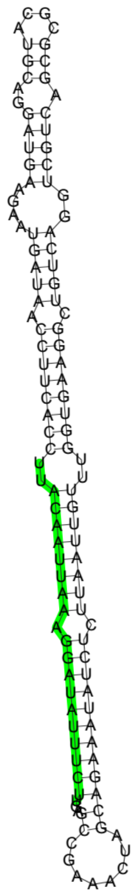

AAGGCGTACCGAACTGAACCGT dre-miR-N62

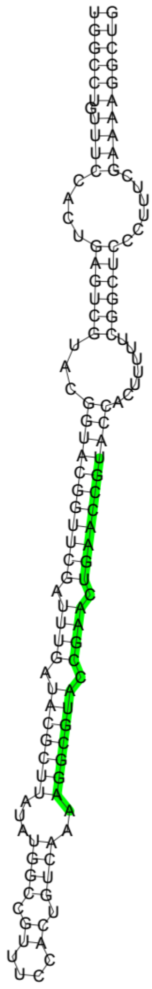

TACACGAGAACACCAGGACACAA dre-miR-N63

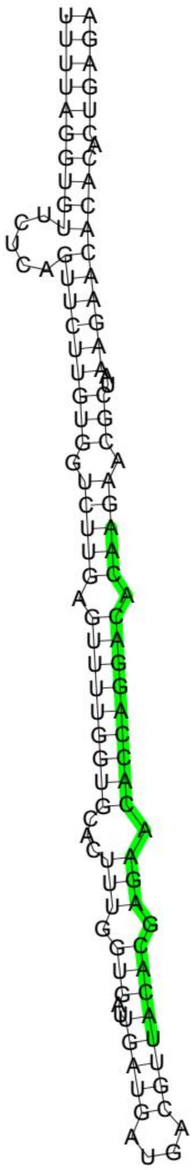

TATGAAAGTCAATGGTTACAC dre-miR-N64

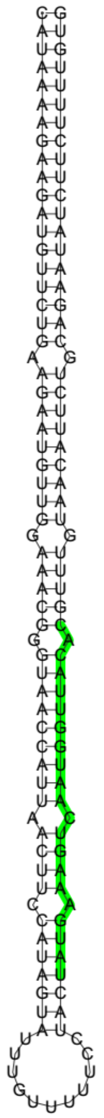

CAGATCCCGGACGAGCCCCCA dre-miR-N65

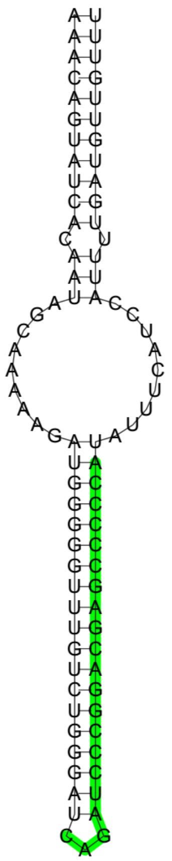

Supplement: S4 Table — (PDF) [file pone.0226905.s007.pdf]
