# Supplementary material for: Identification and characterization of miRNAs involved in cold acclimation of zebrafish ZF4 cells
Source: PLoS One. 2020 Jan 10;15(1):e0226905. doi: 10.1371/journal.pone.0226905 (PMC6953832; doi:10.1371/journal.pone.0226905)
Supplement: Attachment — Submitted filename: Response to reviewers.docx [file pone.0226905.s012.docx]

List of Responses

Dear Editors and Reviewers:

Thanks for your comments concerning our manuscript entitled "**Identification and characterization of miRNAs involved in cold acclimation of zebrafish ZF4 cells**" (Manuscript Number: PONE-D-19-20835R1). These comments are very helpful for improving our data and we have made revision. We would be glad to respond to any further questions and comments. The major corrections and the responds to the reviewer are as following:

**Reviewer 2: The authors didn't show any evidence of the role of microRNAs in fish larvae, so they should change the conclusion that miRNAs are closely involved in cold acclimation in fish in the abstract. Does the miR-16b inhibitor have effects on fish cold acclimation?**

Reply:

**They should change the conclusion that miRNAs are closely involved in cold acclimation in fish in the abstract**

Thank you very much for pointing out this issue. We have corrected this issue in the abstract.

**Does the miR-16b inhibitor have effects on fish cold acclimation?**

It is a very good question. As you pointed out in your comment, for now we do not have the data about the effect of miR-16b inhibitor on fish larvae, so we can not show the effects of miR-16b inhibitor on fish cold acclimation directly. Our ongoing work shows that miR-16b inhibitor microinjection led to increased abnormal development of zebrafish, so those fish larvae are not appropriate for evaluation of the effect of miR-16b inhibitor on fish cold acclimation, because it is hard to attribute observed changes to miR-16b inhibitor or early development defects. Further study of the role of miR-16b in fish may need conditional knockdown of miRNA in zebrafish.

The present data suggested the role of miR-16b in cold acclimation of ZH4 cells based on that miR-16b mimics enhanced cell survival under cold stress when miR-16b inhibitor led to decreased cell viability (Fig. 6B). So we hypothesized that miR-16b inhibitor may have the similar effect on fish cold acclimation.

If you have any suggestion, we will be very glad to hear from you.

Yours sincerely,

Bingshe Han

Bingshe Han, PhD

Building A Room A117

College of Fisheries and Life Science,

Shanghai Ocean University

No.999 Hucheng Ring Road

Shanghai 201306, China

Tel:86-21-61900476/Fax:86-21-61900492

Email: bs-han @shou.edu.cn
